# A low spin manganese(iv) nitride single molecule magnet†
†Electronic supplementary information (ESI) available: Additional NMR, CV, crystallographic, XPS, EPR and magnetic data. CCDC 1471205 and 1471206. For ESI and crystallographic data in CIF or other electronic format see DOI: 10.1039/c6sc01469k


**DOI:** 10.1039/c6sc01469k

**Published:** 2016-06-09

**Authors:** Mei Ding, George E. Cutsail III, Daniel Aravena, Martín Amoza, Mathieu Rouzières, Pierre Dechambenoit, Yaroslav Losovyj, Maren Pink, Eliseo Ruiz, Rodolphe Clérac, Jeremy M. Smith

**Affiliations:** a Department of Chemistry , Indiana University , 800 E. Kirkwood Ave. , Bloomington , IN 47405 , USA . Email: smith962@indiana.edu; b Department of Chemistry , Northwestern University , 2145 Sheridan Road , Evanston , IL 60208 , USA; c Departamento de Química de los Materiales , Facultad de Química y Biología , Universidad de Santiago de Chile (USACH) , Casilla 40, Correo 33 , Santiago , Chile; d Departament de Química Inorgànica , Institut de Recerca de Química Teòrica i Computacional , Universitat de Barcelona , Diagonal 645 , Barcelona , 08028 Spain . Email: eliseo.ruiz@qi.ub.es; e CNRS , CRPP , UPR 8641 , F-33600 Pessac , France . Email: clerac@crpp-bordeaux.cnrs.fr; f Univ. Bordeaux , CRPP , UPR 8641 , F-33600 Pessac , France

## Abstract

Structural, spectroscopic and magnetic methods have been used to characterize the tris(carbene)borate compound PhB(MesIm)_3_Mn≡N as a four-coordinate manganese(iv) complex with a low spin (*S* = 1/2) configuration. The slow relaxation of the magnetization in this complex, *i.e.* its single-molecule magnet (SMM) properties, is revealed under an applied dc field. Multireference quantum mechanical calculations indicate that this SMM behavior originates from an anisotropic ground doublet stabilized by spin–orbit coupling. Consistent theoretical and experiment data show that the resulting magnetization dynamics in this system is dominated by ground state quantum tunneling, while its temperature dependence is influenced by Raman relaxation.

## Abstract 2

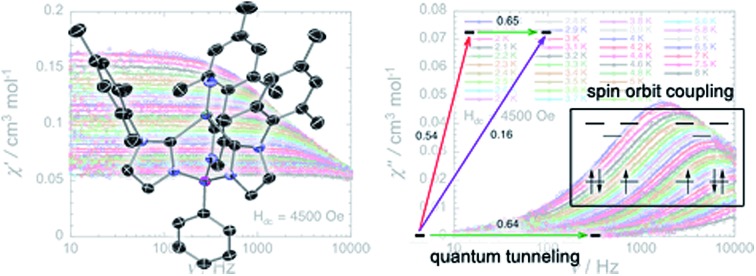
Structural, spectroscopic and magnetic methods have been used to characterize the tris(carbene)borate compound PhB(MesIm)_3_Mn≡N as a four-coordinate manganese(iv) complex with a low spin (*S* = 1/2) configuration.

## Introduction

Since the discovery of a four-coordinate iron(ii) complex displaying SMM behaviour,^1^ multiple examples of mononuclear d-block SMMs have been reported.^2^ In most of these systems, the magnet-like behaviour (*i.e.* their slow dynamics of the magnetization) was described by an Orbach mechanism involving an energy barrier to spin reversal (*Δ*) created by an uniaxial Ising-like magnetic anisotropy (*D*) acting on a high spin ground state (*S*_T_).^3^ Specifically: *Δ* = |*D*|*S*_T_^2^ for integer spins and *Δ* = |*D*|(*S*_T_^2^ – 1/4) for half-integer spins (with *H* = *DS*_*z*_^2^).^4^ With appropriate ligand design, spin–orbit coupling can be used to create a significant uniaxial anisotropy, resulting in large SMM barriers despite the relatively small *S*_T_ associated with mononuclear d-block complexes.^5^ In the case of f-block complexes, spin–orbit coupling is strong and magnetic anisotropy results from crystal field splitting of the total angular momentum (*J*) ground states. Strong spin–orbit coupling can lead to SMM properties in complexes having f^1^ electron configurations. For example, the SMM behaviour of the 5f^1^ U(v) complex, (tren^TIPS^)U(O) (tren^TIPS^ = {N(CH_2_CH_2_NSi^i^Pr_3_)_3_}^3–^) has been attributed to an energy gap between the *M*_*J*_ = ±3/2 ground Kramers doublet and the lowest-lying excited Kramers doublet (either *M*_*J*_ = ±1/2 or *M*_*J*_ = ±5/2).^6^ The SMM properties of 4f^1^ Ce(iii) complexes have been similarly rationalized.^7^ In the case of d-block complexes, there is an intriguing report of a d^9^ SMM, [Ni(6-Mes)_2_]^+^ (6-Mes = 1,3-bis(2,4,6-trimethylphenyl)-3,4,5,6-tetrahydropyrimidin-2-ylidene),^8^ although the origin of the barrier for relaxation of the magnetization was not investigated in detail. A more comprehensive investigation of trigonal planar *S* = 1/2 Ni(i) complexes attributed the observed SMM properties to direct and Raman processes.^9^ Indeed the origin of the magnetization dynamics in these and other *S* = 1/2 SMM systems is often difficult to establish as it can be induced by different mechanisms (Orbach, quantum tunnelling, Raman, direct, phonon-bottleneck-limited direct, *etc.*),^3^,^10^ which are indeed often in intimate competition at a given temperature and applied magnetic field.^9^,^11^

**Fig. 1:**
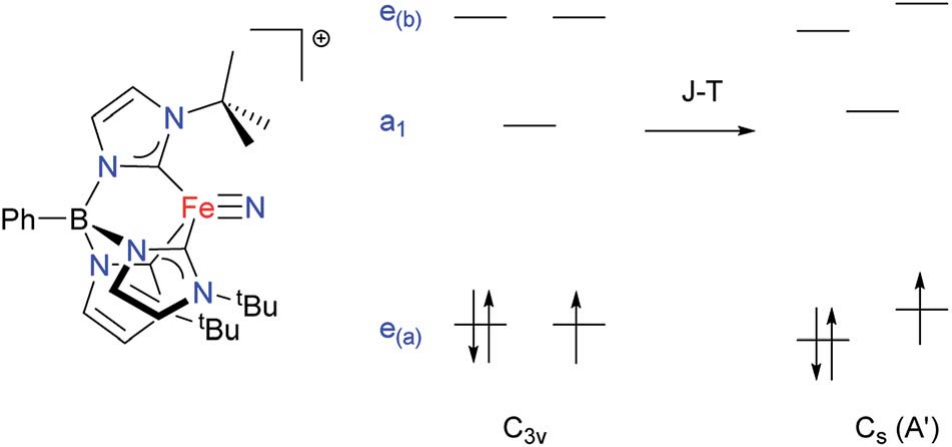
Qualitative illustration of the effect of the Jahn–Teller distortion on the d-orbital splitting in a four-coordinate Fe(v) nitride complex. Due to e–e-mixing, the extent of splitting need not be the same for both sets of e levels.16*b*

Some of us have been investigating the properties of transition metal complexes with strongly donating tris(carbene)borate ligands.^12^,^13^ In addition to stabilizing metal–ligand multiple bonds,^14^ the three-fold symmetric environment induced by these ligands may also be used to create complexes with significant uniaxial anisotropy. This anisotropy leads to slow relaxation of the magnetization in certain high spin iron(ii) tris(carbene)borate complexes.^15^ During the course of these studies, we reported the low spin (*S*_T_ = 1/2) Fe(v) complex, [PhB(^*t*^BuIm)_3_Fe≡N]^+^ (Fig. 1).^16^ Detailed spectroscopic and computational investigations into the electronic structure of this complex reveals that it undergoes a quadratic Jahn–Teller distortion and significant e–e mixing that lowers the idealized molecular symmetry but does not completely quench spin–orbit coupling.16*b*

Building from this work, we report in this contribution the synthesis, characterization, spectroscopic and magnetic properties of the isoelectronic Mn(iv) nitride, PhB(MesIm)_3_Mn≡N (PhB(MesIm)_3_^–^ = phenyltris(3-mesitylimidazol-2-ylidene)borato) which shows similar structural and spectroscopic properties to the Fe(v) complex. Magnetic measurements reveal that this new manganese complex shows slow relaxation of its magnetization, which is unexpected for a low spin (*S*_T_ = 1/2) d^3^ configuration. A combined approach using a detailed experimental study of the relaxation time (in temperature and dc field) and electronic structure theory has been used to delineate the origin of the observed magnetization dynamics in this new SMM.

## Experimental

### General considerations

All manipulations were performed under a nitrogen atmosphere by standard Schlenk techniques or in an MBraun Labmaster glovebox. Glassware was dried at 150 °C overnight. Diethyl ether, *n*-pentane and tetrahydrofuran were purified by the Glass Contour solvent purification system. Deuterated benzene was first dried with CaH_2_, then over Na/benzophenone, and then vacuum transferred into a storage container. Before use, an aliquot of each solvent was tested with a drop of sodium benzophenone ketyl in THF solution. The tris(carbene)borate ligand precursor, PhB(MesImH)_3_OTf_2_, was prepared according to a literature procedure.^13^^1^H NMR data were recorded on a Varian Inova 400 MHz spectrometer at 20 °C. Resonances in the ^1^H NMR spectra are referenced to residual C_6_D_5_H at *δ* = 7.16 ppm. IR spectra were recorded on a Perkin Elmer Spectrum Two spectrometer in THF solution. Cyclic voltammograms were measured using a CH Instruments Model 600B Series Electrochemical Analyzer/workstation in a glovebox with a glassy carbon working electrode. Elemental analysis data were collected by Midwest Microlab, LLC (Indianapolis, IN).

### Synthesis of complexes

#### Preparation of PhB(MesIm)_3_Mn^II^Cl (**1**)

Lithium diisopropylamide (153 mg, 0.46 mmol) was added to a precooled slurry of PhB(MesImH)_3_(OTf)_2_ (437 mg, 1.43 mmol) in Et_2_O (50 mL) at –78 °C. The resulting mixture was stirred at –78 °C for 15 min and then slowly warmed to room temperature. After stirring until the reaction mixture became golden yellow, the solvent was removed *in vacuo*. Tetrahydrofuran (15 mL) was added to the resulting yellow solid, followed by MnCl_2_ (70 mg, 0.56 mmol). The reaction was stirred at room temperature overnight and then dried under vacuum. After washing with Et_2_O and drying under vacuum, the product was obtained (241 mg, yield 71% based on PhB(MesImH)_3_(OTf)_2_) as white solid. Colorless crystals were obtained by diffusion of pentane into a THF solution of the product at –35 °C. *μ*_eff_ = 6.1(3) *μ*_B_ [*χT* = 4.6(1) cm^3^ K mol^–1^]. Elemental analysis calcd for C_42_H_44_BMnCl: (%) C 68.79, H 6.04, N 11.45 found (%) C 68.70, H 6.04, N 11.39.

#### Preparation of PhB(MesIm)_3_Mn^IV^≡N (**2**)

A 250 mL quartz round-bottom-flask was charged with **1** (333 mg, 0.45 mmol), NaN_3_ (146 mg, 2.25 mmol) and THF (100 mL). The mixture was stirred overnight under UV irradiation to yield a yellow solution. The solvent was removed *in vacuo*. Minor impurities were removed by washing with Et_2_O. The remaining solid was extracted into THF and filtered through Celite to yield a yellow solution. The solvent was removed *in vacuo* to afford a yellow solid (201 mg, 56% based on PhB(MesIm)_3_MnCl). X-Ray quality crystals were obtained by the slow diffusion of *n*-pentane into a THF solution of the product at –35 °C. ^1^H NMR (400 MHz, C_6_D_6_): *δ* 12.8 (2H, *o*/*m*-C_6_H_5_); 10.8 (3H, Im-H); 9.3 (2H, *o*/*m*-C_6_H_5_); 8.9 (1H, *p*-C_6_H_5_); 7.0 (6H, Mes *m*-H); 2.7 (9H, Mes *p*-CH_3_); –3.2 (18H, Mes *o*-CH_3_); –11.9 (3H, Im-H). Elemental analysis calcd for C_42_H_44_BMnN_7_·0.5C_4_H_8_O (%) C 71.35, H 6.53, N 13.24; found (%) C 70.56, H 6.51, N 13.32.

### Single-crystal X-ray diffraction

Complex **1** was measured using a Bruker APEX II Kappa Duo diffractometer equipped with an APEX II detector at 150(2) K. Complex **2** was investigated with synchrotron radiation at 100(2) K at the ChemMatCARS 15IDB beamline at the Advanced Photon Source at Argonne National Lab, Chicago. Additional details of the data collection and refinement are included in the ESI.†

### Electron paramagnetic resonance (EPR)

Continuous-wave (CW) X-band (9.32 GHz) EPR spectra of **1** were collected on a modified Bruker ESP-300 spectrometer with 100 kHz field modulation (4 G modulation amplitude) at 20 K through the utilization of an Oxford Instruments liquid helium flow cryostat. Simulations of EPR spectra were performed using the MATLAB EasySpin (v4.5) toolbox (; http://easyspin.org).^17^

### Magnetic susceptibility measurements

The magnetic measurements were carried out with the use of Quantum Design MPMS-XL SQUID magnetometer and PPMS-9 susceptometer. These instruments work between 1.8 and 400 K with applied dc fields ranging from –7 to 7 T (MPMS).

Measurements were performed on a polycrystalline samples of **2** (17.7, 19, 3.2 and 4.5 mg) sealed in a polyethylene bag (3 × 0.5 × 0.02 cm; typical 20 to 40 mg) and covered with mineral oil or directly in their frozen THF mother liquor within a sealed straw to prevent desolvation of the solid. Only experiments done with **2** maintained in frozen mother liquor and prepared under nitrogen atmosphere led to reproducible dc and ac magnetic data. No evaporation of the mother liquor was observed during these measurements. The mass of the sample was determined after the measurements and subsequent mother liquor evaporation. Prior to the experiments, the field-dependent magnetization was measured at 100 K in order to confirm the absence of any bulk ferromagnetic impurities. Ac susceptibility measurements were made with an oscillating field of 1 to 6 Oe with a frequency from 10 to 10 000 Hz (PPMS). The magnetic data were corrected for the sample holder, mineral oil, mother liquor and the intrinsic diamagnetic contributions.

### X-ray photoelectron spectroscopy (XPS)

XPS experiments were performed using PHI *Versa Probe II* instrument equipped with monochromatic Al K(alpha) source. The X-ray power of 50 W at 15 kV was used for 200 micron beam size. The instrument work function was calibrated to give a binding energy (BE) of 84.0 eV for Au 4f_7/2_ line for metallic gold and the spectrometer dispersion was adjusted to give BEs of 284.8, 932.7 and 368.3 eV for the C 1s line of adventitious (aliphatic) carbon presented on the non-sputtered samples, Cu 2p_3/2_ and Ag 3d_5/2_ photoemission lines, respectively. The PHI dual charge compensation system was used on all samples. XPS spectra with the energy step of 0.1 eV were recorded using software *SmartSoft-XPS* v2.0 and processed using PHI *MultiPack* v9.0 at the pass energies of 46.95, 23.5, 11.75 eV for Mn 2p and Mn 3s, for N 1s, and for C 1s regions, respectively. Peaks were fitted using GL line shapes, *i.e.*, a combination of Gaussians and Lorentzians with 0–50% Lorentzian content. Shirley background was used for curve-fitting.

### *Ab initio* calculations

Electronic structure calculations were performed using the ORCA 3.0.3 software package and MOLCAS 8.0.^18^ Energies, wavefunctions and spin-Hamiltonian parameters for full and model complexes were calculated by the CASSCF methodology. The spin–orbit effects were included in both programs using quasi-degenerate perturbation theory (QDPT) in ORCA and restricted active space state interaction (RASSI) approach with MOLCAS program. The def2-TZVP basis set19a,b and ANO-RCC19c,d basis were employed with ORCA and MOLCAS, respectively. Such methods comprise two steps: (i) a CASSCF calculation is performed to obtain the non-relativistic states and energies of the system and (ii) state mixing by the Spin–Orbit Coupling (SOC) operator. Dynamical correlation was introduced by the N-electron valence perturbation theory (NEVPT2).^20^ Energies for the d orbitals were obtained from the *ab initio* ligand field theory (AILFT) approach.21*a* In a nutshell, the AILFT approach allows for the extraction of ligand field and Racah parameters from a one-to-one mapping of the matrix elements of a model ligand field matrix to a CI matrix obtained from electronic structure methods (in this case, the CI matrix from a CASSCF(3,5) calculation). Numerical values for the parameters are obtained from least-squares fit of the CASSCF matrix elements and orbital energies can be calculated by diagonalization of the ligand field matrix. Further details about the CASSCF + QDPT approach, the AILFT method and its applications to problems in molecular magnetism have been previously described.21*b*

## Results and discussion

### Synthesis and characterization

**Fig. 2:**
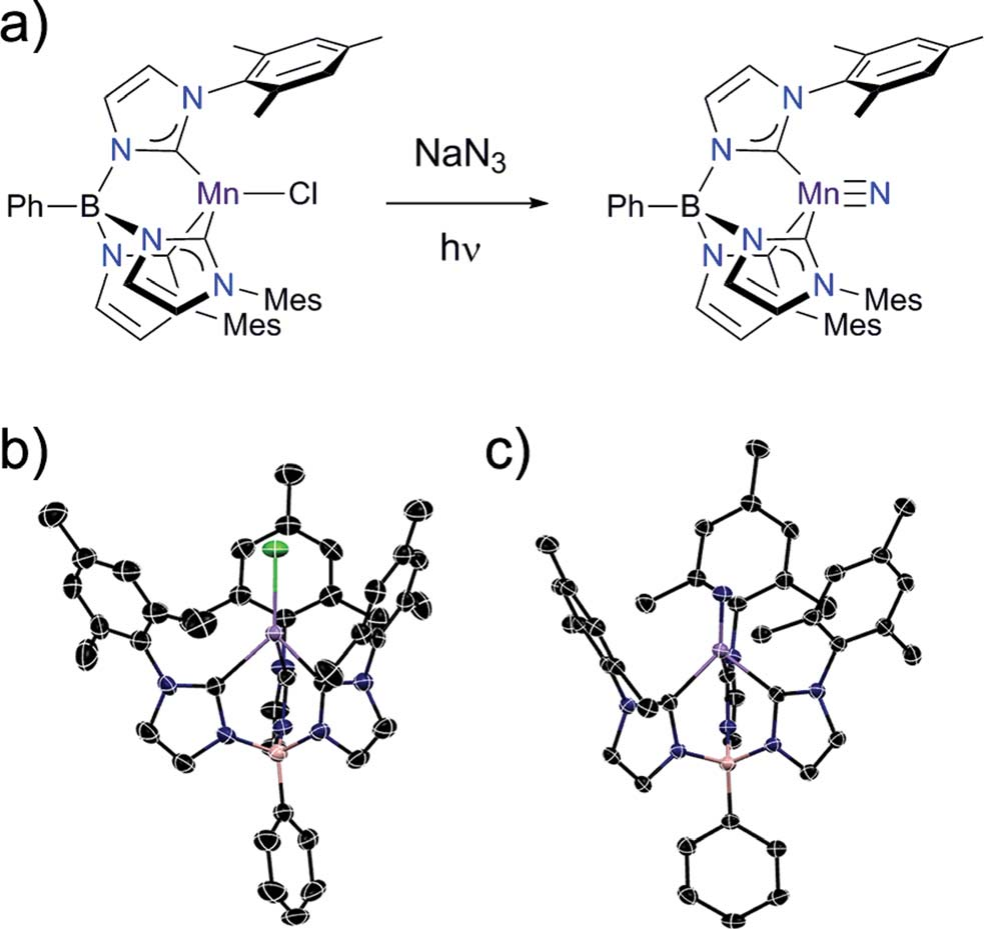
(a) Synthesis of PhB(MesIm)_3_Mn≡N (**2**) and X-ray crystal structures of (b) PhB(MesIm)_3_Mn^II^–Cl (**1**), and (c) PhB(MesIm)_3_Mn^IV^≡N (**2**) with thermal ellipsoids shown at 50% probability; H atoms are omitted for clarity. Black, blue, lilac, pink and green ellipsoids represent C, N, Mn, B and Cl atoms, respectively.

The manganese nitride complex, PhB(MesIm)_3_Mn≡N (**2**) is accessible by the same synthetic pathway used to prepare the related Fe(iv) nitrides (Fig. 2a).^22^ Specifically, irradiating a solution of the high spin Mn(ii) complex PhB(MesIm)_3_MnCl (**1**) (Fig. 2b) in the presence of NaN_3_ provides **2** as a yellow solid following workup. Structural and spectroscopic methods (detailed below) reveal **2** to be a four-coordinate Mn(iv) nitride complex with a low spin (*S*_T_ = 1/2) d^3^ electron configuration that is subject to a Jahn–Teller distortion. The molecular structure of **2** has been determined by single crystal X-ray diffraction (Table S1†), revealing a four-coordinate manganese nitride complex supported by the tripodal tris(carbene)borate ligand (Fig. 2c), that crystallizes with interstitial THF molecules. The asymmetric unit contains one THF and two independent molecules with similar metrical parameters; only one of these will be discussed (Table S2†). The Mn–N (1.523(2) Å) and Mn–C (1.938(2)–2.006(2) Å) distances are slightly longer than the equivalent distances in the related tris(carbene)borate Fe(iv) nitrides,^22^ likely due to the larger ionic radius of the Mn(iv) centre. The manganese ion lies *ca.* 0.1 Å out of the plane defined by the carbon atoms of the tris(carbene)borate ligand, which is similar to the equivalent distance observed in the iron analogues. Similarly to the isoelectronic [PhB(^*t*^BuIm)_3_Fe^V^≡N]^+^ complex,^16^ the Jahn–Teller distortion is manifested in the B–Mn–N vector bending away from 180° (B–Mn–N = 174.7°). While many of the metrical parameters are similar, there are some key structural differences between **2** and the related Mn(iv) nitride [(TIMEN^xyl^)Mn≡N]^+^ (TIMEN^xyl^ = tris[2-(3-xylylimidazol-2-ylidene)ethyl]-amine)^23^ (Table 1). The most notable structural differences relate to how the Jahn–Teller distortion is manifested (Fig. S3†). In the case of [(TIMEN^xyl^)Mn≡N]^+^, which has a relatively flexible tris(carbene)amine ligand, significant elongation of one Mn–C bond (by 0.15 Å) occurs to lower the local symmetry at the Mn site. The greater rigidity of the tris(carbene)borate ligand in **2** evidently hinders such a distortion, and all Mn–C distances are similar in length. Instead, the B–Mn–N angle in **1** is bent away from 180° (B–Mn–N = 174.7°), whereas the equivalent angle in [(TIMEN^xyl^)Mn≡N]^+^ is almost linear (179.4°).

**Table 1:** Comparative structural and spectroscopic data for low spin Mn(iv) nitride complexes

Complex	Mn–N (Å)	Mn–C (Å)	E–Mn–N^*a*^ (°)	EPR	*E* (V)
PhB(MesIm)_3_Mn≡N (**2**)	1.523(2)	1.938(2)	174.7	*g*_1_ = 2.35	–0.82 V^*b*^
		1.956(2)		*g*_2_ = 1.973	–2.30 V
		2.006(2)		*g*_3_ = 1.965	
(TIMEN^xyl^)Mn≡N^+^	1.524(3)	1.932(6)	179.4	*g*_1_ = 2.22	–1.1 V
		1.990(5)		*g*_2_ = 1.98	–2.4 V
		2.103(5)		*g*_3_ = 1.97	

^*a*^E = B for **2**, E = N for (TIMEN^xyl^)Mn≡N^+^.

^*b*^Oxidation of **2** is irreversible.

Complex **2** has also been spectroscopically characterized. The solution ^1^H NMR spectrum reveals eight paramagnetically-shifted resonances with relative integration appropriate for a three-fold symmetric complex. The solution magnetic moment, as determined by the Evans' method (*μ*_eff_ = 2.2(3) *μ*_B_; *χT* = 0.6(1) cm^3^ K mol^–1^), is consistent with a single unpaired electron and unquenched spin–orbit coupling seen in the solid state (see below).

The redox characteristics of **2** have been investigated by cyclic voltammetry. As with the structural data, interesting differences with [(TIMEN^xyl^)Mn≡N]^+^ are observed (Table 1), likely stemming from the relative flexibilities of the two tris(carbene) ligands. Thus, while both **2** and [(TIMEN^xyl^)Mn≡N]^+^ can be reversibly reduced on the CV timescale, only the latter can be oxidized to Mn(v).^23^ The stability of the Mn(v) state for the TIMEN^xyl^ ligand is in part due to the ability of apical bridgehead nitrogen atom of this ligand to bind to Mn in this higher oxidation state, forming a five-coordinate complex. Such additional stabilization is not possible with the tris(carbene)borate ligand.

### Electron paramagnetic resonance

**Fig. 3:**
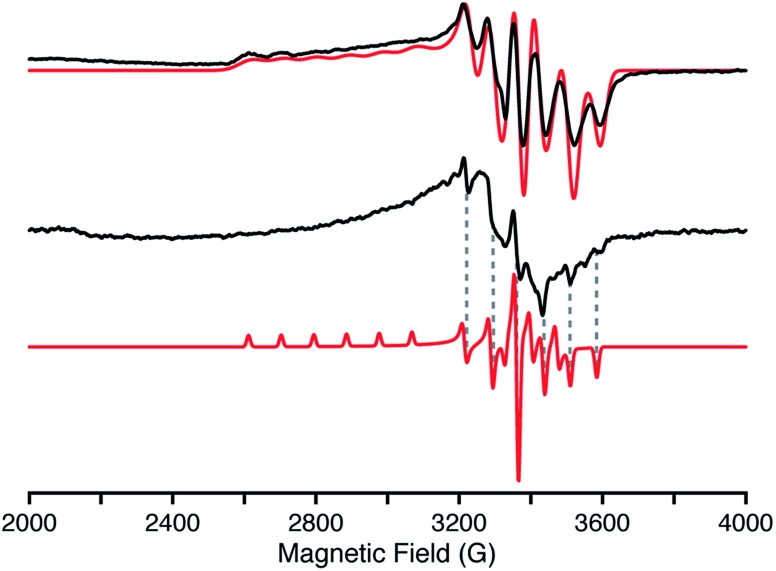
X-Band (9.37 GHz) continuous-wave EPR of **2** in solution (top) and suspended powder (middle) with simulations (red) collected at 20 K with 100 kHz field modulation (4 G modulation amplitude). The solution exhibits an axial EPR and is simulated by the following parameters: **g** = [*g*_1_, *g*_2_, *g*_3_] = [2.35, 1.973, 1.965]; **A**(^55^Mn) = [*A*_1_, *A*_2_, *A*_3_] = [300, 74, 202] MHz; EPR lw = [250, 85, 85] MHz. The suspended powder (slurry) exhibits very anisotropic EPR linewidths of the three conical *g*-values. An EPR simulation with isotropic linewidths (25 MHz) is shown (bottom) as a visual aid to the reader to identify the *A*_3_ hyperfine features (dashed lines).

More detailed insights into the electronic structure of **2** have been obtained from EPR spectroscopy. The frozen solution EPR spectrum (Fig. 3, top) incorporates resolved hyperfine splitting from the Mn(iv), *I* = 5/2, centre. The |*M*_*I*_〉 = |−5/2〉 and | = |–5/2〉 = |−5/2〉 and | and |*M*_*I*_ = |–3/2 = |−3/2〉 manifolds at the low magnetic field edge of manifolds at the low magnetic field edge of *g*_∥_ are well resolved, and simulated with an *A*_1_(^55^Mn) = 300 MHz coupling. The *g*_⊥_ values are slightly split, with anisotropic ^55^Mn hyperfine couplings, as determined by simulation of the EPR spectrum, yielding *g* values (**g** = [*g*_1_, *g*_2_, *g*_3_] = [2.35, 1.973, 1.965]) and ^55^Mn couplings (**A** = [*A*_1_, *A*_2_, *A*_3_] = [300, 74, 202] MHz). The average *g* value, *g*_av_ = [(*g*_1_ + *g*_2_ + *g*_3_)/3] = 2.096, is in agreement with the *g* factor, 2.1(1), determined from the magnetic susceptibility measurements detailed below. The electronic structure of **2** and EPR parameters remarkably resemble those of other low-spin trigonal d^3^ centres (Mn(iv) and Fe(v)) with tris(carbene) ligands.^16^,^23^ A solid powder sample of **2** was also prepared for EPR characterization by suspending the solid in pentane to form a slurry. The X-band EPR spectrum of this slurry (Fig. 3, middle) is similar to that observed in solution (Fig. 3, top). The same *g*_⊥_ ∼ 1.97 feature is observed, although with anisotropic line widths. The *g*_∥_ (*g*_1_) feature is too broad and not observed, however, the *A*_3_^55^Mn hyperfine splitting of 204 MHz is distinctly observed in the EPR spectrum of the slurry (Fig. 3, bottom dashed lines). The line widths of the *A*_2_ hyperfine lines are noticeably broader than *A*_3_. Therefore, the *g*_2_, *g*_3_ and ^55^Mn hyperfine (*A*_2_, *A*_3_) parameters of the slurry sample match those observed for the solution. EPR spectra of this slurry collected at various temperatures (3.6 to 20 K) exhibit only the *S* = ½ Mn(iv) complex identifiable by the ^55^Mn hyperfine structure (see ESI†). In short, the electronic characteristics of the d^3^ Mn(iv) nitride are the same in both solution and the solid state.

### X-ray photoelectron spectroscopy

**Fig. 4:**
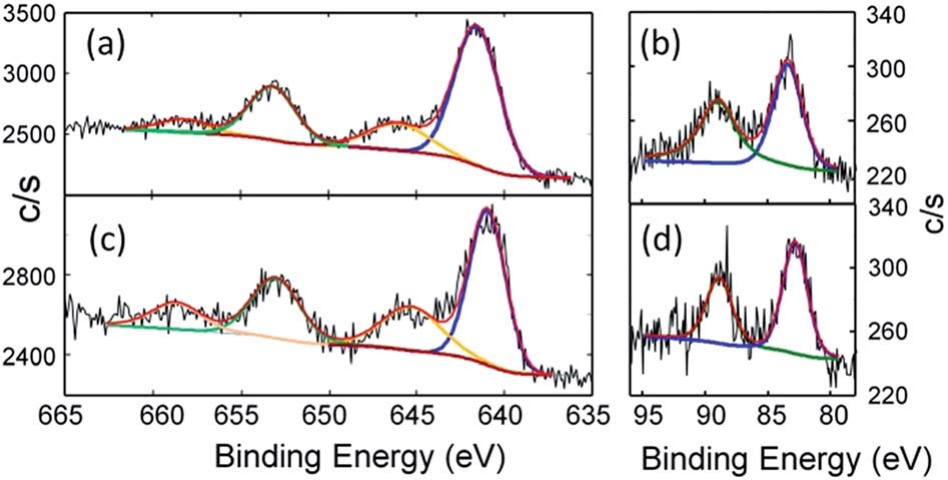
High-resolution Mn 2p spectra of (a) PhB(MesIm)_3_Mn≡N (**2**) and (c) PhB(MesIm)_3_MnCl (**1**). The black line represents the experimental data, the red line shows the fit, and the blue and green lines represent Mn 2p_3/2_ and Mn 2p_1/2_ components, respectively, while the brown line represents shake-up satellites. See Table S3† for fitting parameters. High-resolution Mn 3s spectra of (b) PhB(MesIm)_3_Mn≡N (**2**) and (d) PhB(MesIm)_3_MnCl (**1**). The black line represents the experimental data, the red line shows fit, and the blue and green lines represent Mn 3s split components. See Table S4† for fitting parameters.

The combined structural and spectroscopic data described above indicate the presence of a tetravalent manganese ion in complex **2**. This oxidation state assignment has been confirmed using X-ray Photoelectron Spectroscopy (XPS). The standard position of the 2p_3/2_ peak for the Mn(iv) state is accepted to be in the range from 641.1 to 642.5 eV with the spin–orbit splitting of 11.7 eV between Mn 2p_3/2_ and Mn 2p_1/2_ levels. The measured binding energies of Mn 2p_3/2_ for **2** are well within this range (Fig. 4a, for details see Table S2†). It should be noted that the shape of the Mn 2p transition may be different for samples with the same Mn oxidation state. Thus, for example, a shake-up-like satellite (normally characteristic of Mn^2+^ ions) is observed for MnPO_4_, but not for Mn_2_O_3_, despite the Mn(iii) state of both compounds.^24^ Similar shake-up-like features are observed for our Mn(iv) complex, which clearly are more resolved for the Mn(ii) complex **1** (Fig. 4c) as expected. The feature similar to the shake-up high energy side of the Mn 2p_3/2_ shoulder was also reported for nanoparticles containing Mn(iv) ions in a SnO_2_ matrix.^25^ The Mn 3s spin–orbit split for both samples was also recorded to better distinguish between the 4+ and 2+ oxidation states of Mn. The clear reduction of the value for the spin–orbit split for **2** in comparison to that of **1** (Fig. 4b and d and Table S4†) is consistent with reported in literature values.^26^ We found a measurable difference in the binding energies of N 1s as well as differences in the ratio of the components (Fig. S3 and S4†). The N 1s region for **2** is deconvoluted in a 3 : 3 : 1 ratio, while a 1 : 1 ratio is observed for **1**, as expected. Thus, the XPS data are fully consistent with manganese being in the +IV oxidation state in complex **2**.

In summary, the combined characterization data reveal that **2** is the latest addition to the small but growing family of compounds having a low spin (*S*_T_ = 1/2) d^3^ electron configuration.^16^,^23^,^27^,^28^ It is notable that many of these complexes are supported by ligands that create approximately three-fold symmetric environments.^16^,^23^,^28^ This electronic configuration is susceptible to a Jahn–Teller distortion away from three-fold symmetry. This distortion is most clearly observed in 3d metal complexes, where the nature of the distortion depends on the supporting ligand.

### Magnetic properties

**Fig. 5:**
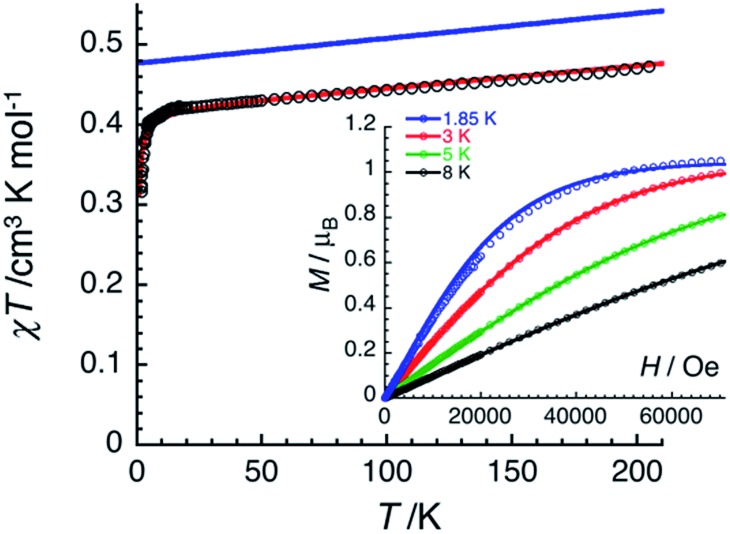
Temperature dependence of the *χT* product at 0.1 T (*χ* is defined as magnetic susceptibility equal to *M*/*H* per mole of **2**). Inset: field dependence of the magnetization below 8 K for **2** (8–200 mT min^–1^). Solid lines are simulations discussed in the text.

The magnetic properties of **2** have been studied by dc and ac techniques. Perfectly reproducible data were obtained when the compound was maintained below 200 K during the measurements and in its THF mother liquor, which prevents loss of solvent from the polycrystalline sample. At 200 K, the *χT* product has a value of 0.47 cm^3^ K mol^–1^ in good agreement with a magnetically isolated low-spin (*S*_T_ = 1/2) Mn(iv) centre (Fig. 5). When lowering the temperature, the *χT* product decreases first almost linearly down to 30 K and then in a more pronounced manner to reach 0.32 cm^3^ K mol^–1^ at 1.85 K. As shown by the electronic structure calculations discussed in the next section, the observed thermal behaviour above 30 K is directly the consequence of the thermal depopulation of the first excited doublet state. As expected, the theoretical *χT vs. T* data calculated using MOLCAS code^18^ (blue line in Fig. 5) compare qualitatively very well with the experimental data (it is worth noting that the higher theoretical *χT* value is due to the larger calculated *g*_av_ value; see Electronic structure calculations section). At lower temperatures and as already detected by EPR (*vide supra*), the marked decrease of the *χT* product reveals the presence of antiferromagnetic interactions between Mn(iv) complexes. These intermolecular interactions were evaluated at –0.6(1) K (*zJ*/*k*_B_) by simulating the experimental data in the frame of the mean-field approximation^29^ applied to the scaled (×0.88) MOLCAS *χT vs. T* values (red line in Fig. 5). The field dependences of the magnetization below 8 K (inset Fig. 5) are also in good agreement with an *S* = 1/2 species (*M* = 1.05 *μ*_B_ at 7 T & 1.85 K). The fit of the experimental data with an *S* = 1/2 Brillouin function confirms an average *g* factor around 2.10(2), which is in perfect agreement with that deduced from EPR (*g*_av_ = 2.096, *vide supra*).

**Fig. 6:**
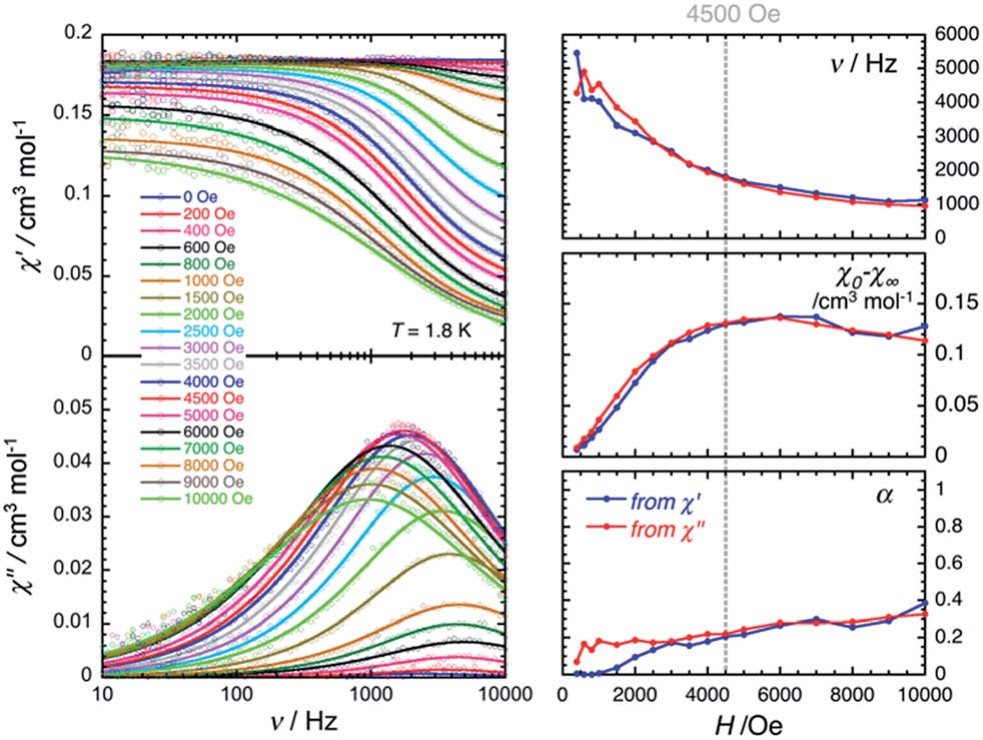
Left part: Frequency dependence of the real (*χ*′, top) and imaginary (*χ*′′, bottom) parts of the ac susceptibility at 1.8 K at different dc fields between 0 and 1 T for a polycrystalline sample of **2**. Solid lines are the best fits of the experimental data to the generalized Debye model.^30^ Right part: Temperature dependence of the magnetic parameters deduced from the fits of the *χ*′ *vs. ν* (blue dots) and *χ*′′ *vs. ν* (red dots) data shown in the left part of the figure using the generalized Debye model^30^ (*ν*: characteristic ac frequency; *χ*_0_ – *χ*_∞_: amplitude of the relaxation mode with *χ*_0_ and *χ*_∞_ being the in-phase ac susceptibilities in the zero and infinite ac frequency limits, respectively; *α*: the distribution of the relaxation). The solid lines are guides for the eyes.

**Fig. 7:**
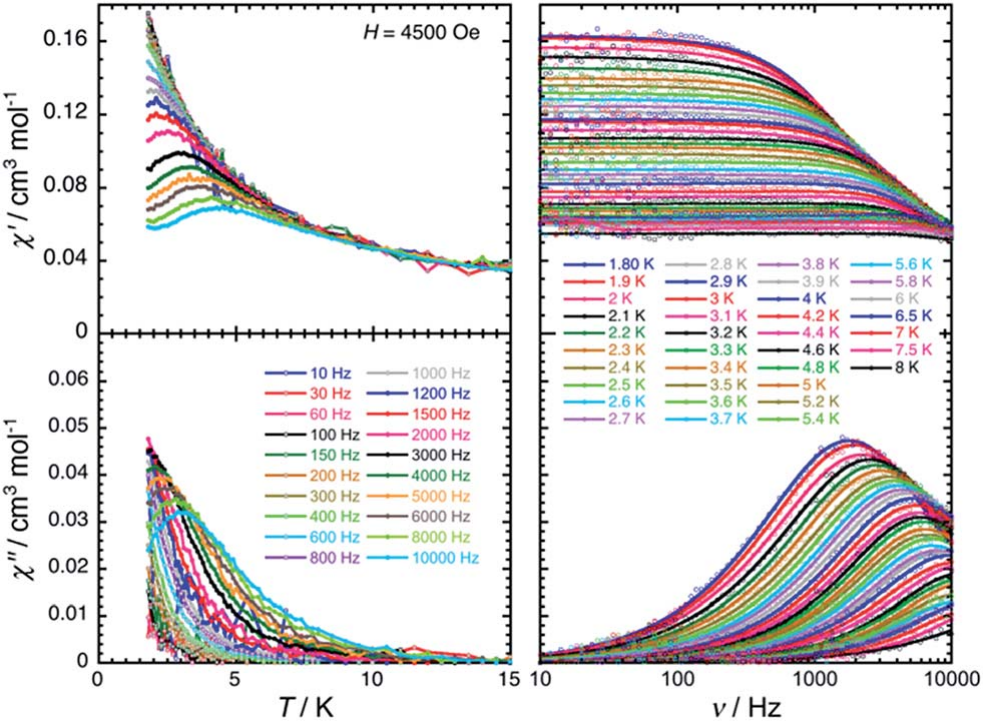
Temperature (left) and frequency (right) dependences of the real (*χ*′, top) and imaginary (*χ*′′, bottom) parts of the ac susceptibility, between 1.8 and 15 K and between 10 and 10 000 Hz respectively, for **2** in a 0.45 T dc field. Solid lines are visual guides on the left part of the figure and are the best fits of the experimental data to the generalized Debye model.

The magnetization dynamics of this manganese nitride complex were probed by ac susceptibility measurements. In the absence of a dc field, the ac data, above 1.8 K and for frequencies up to 10 kHz, display a frequency independent in-phase (*χ*′) susceptibility consistent with the dc susceptibility (Fig. 5), and accordingly do not exhibit any out-of-phase component (*χ*′′). However, application of a dc field leads to the detection of a relaxation process in both components of the ac signal (Fig. 6), revealing the slow dynamics of the magnetization in **2**. The ac signal becomes detectable around 5000 Hz for a dc field of about 200 Oe. At all fields, the *χ*′ *vs. ν* and *χ*′′ *vs. ν* data can be modelled by a generalized Debye model^30^ (Fig. 6) with a small *α* coefficient (<0.4) indicating a weak distribution of the relaxation time (*τ*) and thus a relaxation mode that is dominated by a single relaxation process. The characteristic frequency of this relaxation mode continuously decreases when applying higher fields (to about 1000 Hz at 1 T) while the amplitude of the mode (*χ*_0_ – *χ*_∞_) exhibits a maximum around 0.45 T (Fig. 6). For this particular dc field, the temperature dependence of the ac susceptibility was studied as shown in Fig. 7. At all temperatures, the *χ*′ *vs. ν* and *χ*′′ *vs. ν* data can also be modelled by a generalized Debye model^30^ (Fig. 7 and S7†) allowing an estimation of the temperature dependence of the relaxation time at 0.45 T (Fig. S8†). As conventionally admitted, the exponential increase of the relaxation time (*i.e.* it follows an Arrhenius law) suggests the presence of a thermally activated (Orbach) process of relaxation with a pre-exponential factor, *τ*_0_, of 5(1) × 10^–6^ s and an energy gap of only 5.1(5) K (3.5 cm^–1^). While the origin of the relaxation process will be discussed in more detail below, it is important at this stage to note the unusually small energy barrier and the large value of *τ*_0_ (at least 4 orders of magnitude larger than expected for typical vibrations of the network which govern the Orbach reversal of magnetization).^3^

### Electronic structure calculations

The magnetic properties of the low-lying states of **2** were further analysed by means of an *ab initio* multireference methodology. A symmetrized model complex was first studied to obtain a qualitative description of the ground state nature of **2** and then these conclusions were corroborated by calculations of the full complex.

**Fig. 8:**
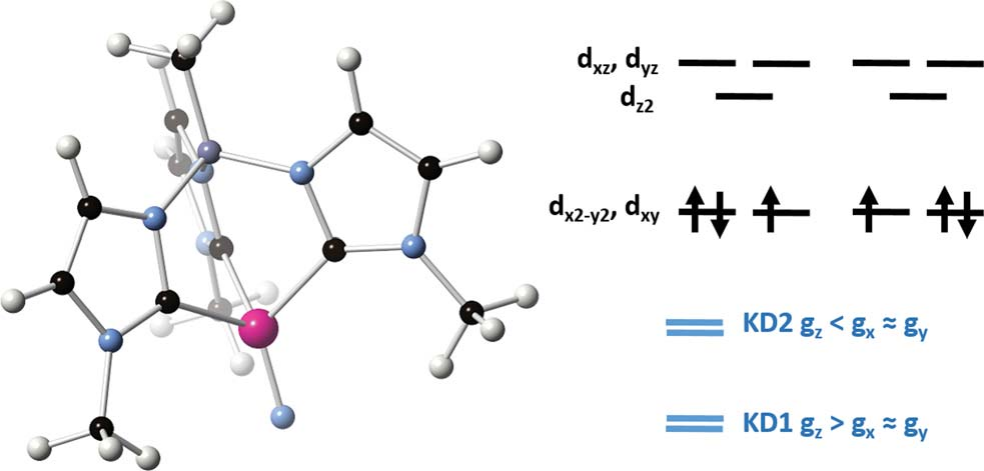
Left: Model complex for **2** used in the electronic structure calculations. Color code: Mn (magenta); N (light blue); C (black); B (purple); H (white). Right, top: Main orbital configurations contributing to the ground state. Right, bottom: Relation between components of the *g* tensor of the first two Kramers' doublets (KD1 and KD2).

**Fig. 9:**
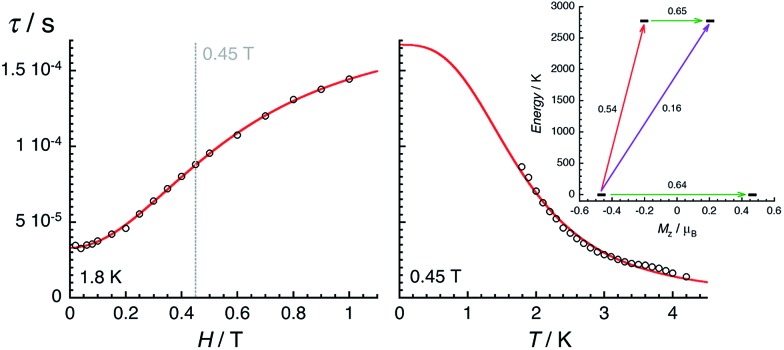
Field (left, at 1.8 K) and temperature (right, at 0.45 T) dependences of the average relaxation time for **2** estimated from the Fig. 6 and 7. The red lines are the best fits obtained with the theoretical approach developed in the text. Inset: lowest two Kramers doublets and *ab initio* computed relaxation mechanism with the MOLCAS code (CASSCF + RASSI level). The thick black lines are Kramers doublets shown as a function of their magnetic moment, *M*_*z*_, along the main anisotropy axis (*z*). The green arrows correspond to the quantum tunnelling mechanism (QTM) of ground and first excited states while purple arrow shows the hypothetical Orbach relaxation process. The red arrow indicates the transition between the ground and first KDs. The values close to the arrows indicate the matrix elements of the transition magnetic moments (above 0.1, an efficient spin relaxation mechanism is expected). Thus, this figure highlights that the QTM through the Kramers doublet ground state is dominating the relaxation process at low temperatures.

The model complex was constructed from its original geometry, where the aryl groups were replaced with methyl substituents, symmetrizing the structure to the *C*_3v_ group. Initial CASSCF(3,5) calculations for the model system using the ORCA code indicate the following orbital sequence (obtained with the AILFT theory, see *ab initio* calculations section): d_*xy*_ and d_*x*^2^–*y*^2^_ at a reference energy (*i.e.* 0.0 cm^–1^), d_*z*^2^_ at 31 000 cm^–1^ and (d_*xy*_, d_*xz*_) at 32 500 cm^–1^ (which is equivalent to the orbital diagram of Fig. 1; see also Fig. 8). Although this orbital diagram appears to be reasonable, the limitations of this reduced active space are evident in the swapping of the ground state wavefunction due to the inclusion of dynamical correlation (NEVPT2) and the prediction of a quartet state as the ground level. The addition of the *σ* and 2π orbitals of the N^3–^ ligand in a CASSCF(9,8) leads to the correct spin state ordering and a NEVPT2 correction that preserves the ground state for the model structure. The lower energy orbitals in the CASSCF(9,8) calculations are still (d_*xy*_, d_*x*^2^–*y*^2^_), with a doubly degenerate ground state that corresponds predominantly (81% weight in both wavefunctions) to the 
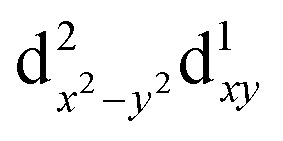
 and 
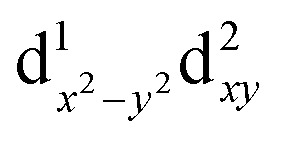
 configurations. The next excited state is 7300 cm^–1^ higher in energy (10 100 cm^–1^ in NEVPT2) and is not relevant for discussing the SMM properties of **2**. Thus, magnetic anisotropy in this system emerges from the quantum mixing of the degenerate ground state by the spin–orbit coupling (SOC), given that the d_*x*^2^–*y*^2^_ and d_*xy*_ orbitals are connected by the *z* component of the angular momentum operator.2b,11b,^31^ In this way, two strongly anisotropic Kramers' doublets are formed from the first two *S* = 1/2 states, separated by 470 cm^–1^ (676 K). The ground doublet of the model system presents a markedly uniaxial *g* tensor with *g*_*z*_ = 5.15, *g*_*x*_ = *g*_*y*_ = 0.15. This strong anisotropy is significantly reduced in the full system due to the deviations from trigonal symmetry that breaks the degeneracy between d_*x*^2^–*y*^2^_ and d_*xy*_ orbitals, partially quenching the SOC mixing. In the full complex, the calculated ground state is split to an energy difference of 2103 cm^–1^ (CASSCF(9,8) calculation including only doublets). This splitting leads to a marked decrease of the *g* tensor anisotropy of the ground doublet to values of *g*_*x*_ = 1.940, *g*_*y*_ = 1.942 and *g*_*z*_ = 2.674, yielding a *g*_av_ of 2.185, in satisfactory agreement with the values obtained for magnetization and EPR measurements. Equivalent CASSCF + RASSI calculations performed with MOLCAS code provide similar values with a first excited Kramers doublet at 1932 cm^–1^ (2800 K) and *g*_*x*_ = 1.927, *g*_*y*_ = 1.933 and *g*_*z*_ = 2.790 values (*i.e. g*_av_ = 2.217; as mentioned above the calculated *g*_av_ values is systematically slightly larger than the experimental one, around 2.1, which explains the difference between experimental and calculated magnetic susceptibility exemplified in Fig. 5). This CASSCF + RASSI approach was also used to estimate the possible relaxation mechanisms considering the two lowest Kramers doublets (Fig. 9 inset). As expected due to their large energy separation, thermally activated mechanisms of relaxation, *e.g.* Orbach, cannot be relevant at low temperatures. Thus, the magnetic dynamics in **2** should only involve the ground Kramers' doublet, allowing possible quantum tunnelling (QTM), direct and Raman mechanisms.

### Discussion on the origin of the magnetization relaxation

The electronic structure calculations discussed in the previous section lead to unambiguous conclusions on the origin of the slow dynamics of the magnetization in **2**, which (i) should be dominated by QTM, direct and/or Raman mechanisms and (ii) cannot involve Orbach processes. With these elements in mind, the experimental relaxation time has been further analysed starting from its field dependence at 1.8 K (Fig. 9 left). At low fields (*μ*_B_*H* ≪ *k*_B_*T*), most of the processes inducing a magnetization relaxation (Raman, Orbach, phonon-bottleneck, *etc.*) are weakly field dependent and thus they have been included as constant, *k*(*T*), in eqn (1).^3^,^11^ On the other hand, the quantum tunnelling of the magnetization is strongly affected by applying even a small magnetic field as illustrated by the first term in eqn (1).^32^,^33^ As shown in Fig. 9 (left part), the experimental relaxation time is extremely well described by this simple approach (eqn (1); with *B*_1_ = 24 800(50) s^–1^, *B*_2_ = 15.6(5) T^–2^ and *k*(*T*) = 5427 s^–1^) confirming the key role of the quantum tunnelling of the ground doublet in the relaxation mechanism, in agreement with the theoretical predictions (Fig. 9 inset). As direct processes are also strongly field dependent (even at low fields),^3^,^9^,^32^ their possible incidence on the magnetization dynamics of this Kramer system was also tested by including an *TH*^4^ term in eqn (1). The fit of the experimental data (Fig. 9, left part) to this more complete model leads systematically to a negligible prefactor of this additional *TH*^4^ term underlying the irrelevance of the direct processes.
1

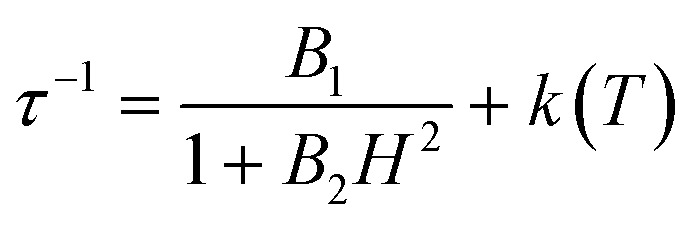




The temperature dependence of the relaxation at 0.45 T was analysed analogously, considering *τ*_QTM_ as a constant (equal to 1.67 × 10^–4^ s from the first term of eqn (1)) and including thermally active processes, which are either thermally activated (Orbach) or following a power law of the temperature for Raman mechanisms (with exponents ranging from 1 to more than 9).^3^,^9^,^11^,^32^

2 *τ*^–1^ = *τ*_QTM_^–1^ + *bT*^*n*^

Remarkably, eqn (2) is able to reproduce the experimental data with a single power law and an exponent (*n*) of 2.93(5) (with *b* = 1105 s^–1^ K^–2.93^ and *τ*_QTM_ fixed at 1.67 × 10^–4^ s). As discussed recently by Sessoli *etal.* for an *S*_T_ = ½ V^IV^ complex,11*d* the exponent value close to 3 suggests the presence of a Raman process involving both acoustic and optical vibrations.^9^ It is worth mentioning that the addition of terms in eqn (2) including different power laws or an exponential function does not significantly improve the modelling of the experimental data shown in Fig. 9. Overall, the combined field and temperature dependence of the relaxation time below 4 K and 1 T confirms the predominance of the quantum tunnelling pathway to relax the magnetization with a characteristic time of *ca.* 2 × 10^–4^ s. Nevertheless, this relaxation mechanism is clearly assisted by Raman processes that rationalize the thermal dependence of the relaxation time.

## Conclusions

Structural and spectroscopic methods reveal that the Mn(iv) complex PhB(MesIm)_3_Mn≡N (**2**) is a rare example of a low spin (*S* = 1/2) d^3^ complex. Its degenerate electron configuration is subject to a Jahn–Teller distortion, which is manifested in **2** by bending of the B–Mn–N vector, similarly to the isoelectronic Fe(v) complex, [PhB(^*t*^BuIm)_3_Fe≡N]^+^.^16^ Electronic structure calculations confirm the role of the spin–orbit coupling to stabilize an anisotropic ground doublet even in presence of the Jahn–Teller distortion. As the first excited doublet lies more than 2000 cm^–1^ above the ground state, SMM properties observed by ac susceptibility measurements cannot rely on an Orbach mechanism and even if the traditional semi-logarithm *τ vs. T*^–1^ presentation of the experimental data could suggest the contrary. A detailed analysis of the field and temperature dependence of the relaxation time supports the theoretical CASSCF + RASSI calculations, and highlights the key role of the quantum tunnelling mechanism in the slow dynamics of the magnetization in this *S* = ½ species. Additionally, the signature of Raman processes could be detected in the thermal variation of the relaxation time. Since theoretically the Jahn–Teller distortion significantly activates the quantum tunnelling mechanism, we anticipate that complexes where the structural distortion is smaller than in **2** will have much larger relaxation times. Investigations aimed at testing this hypothesis are currently in progress.

## Supplementary Material

Supplementary information

Crystal structure data
