# Supplementary material for: A low spin manganese(iv) nitride single molecule magnet
Source: Chem Sci. 2016 Jun 9;7(9):6132–40. doi: 10.1039/c6sc01469k (PMC5058364; doi:10.1039/c6sc01469k)
Supplement: Supplementary file 1 [file SC-007-C6SC01469K-s001.pdf]

Electronic Supplementary Material (ESI) for Chemical Science.
This journal is © The Royal Society of Chemistry 2016

Supporting Information for

A Low Spin Manganese(IV) Nitride Single Molecule Magnet

Mei Ding, George E. Cutsail, III, Daniel Aravena, Martín Amoza, Mathieu Rouzières, Pierre Dechambenoit, Yaroslav Losovyj, Maren Pink, Eliseo Ruiz, Rodolphe Clérac and Jeremy M. Smith

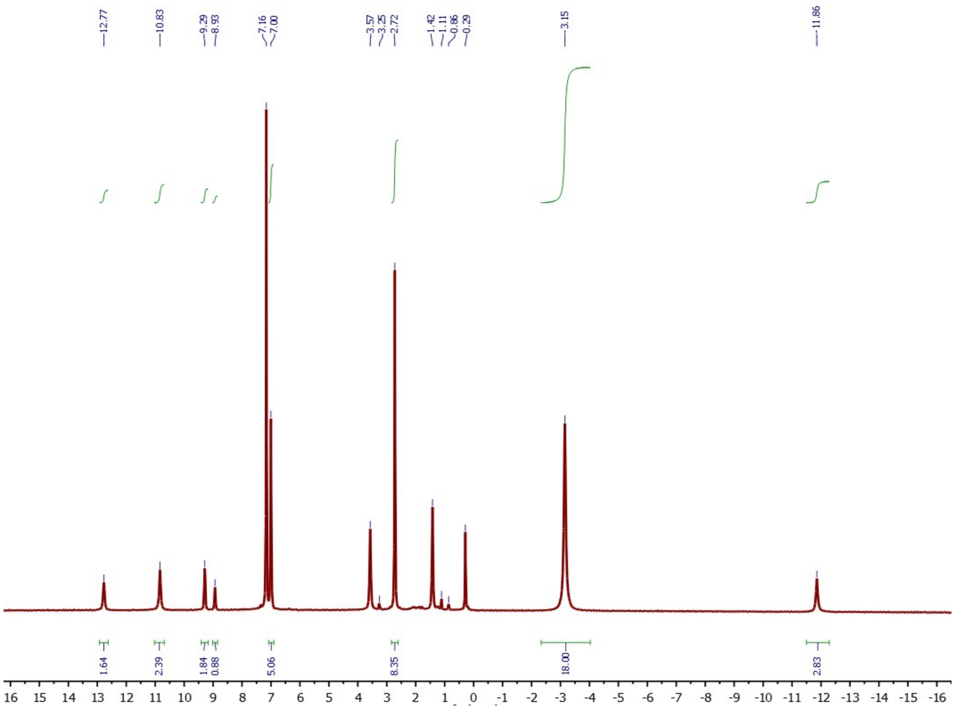

Figure S1. ^{1}H NMR spectrum of **2** (400 MHz, C_6D_6, 20 °C).

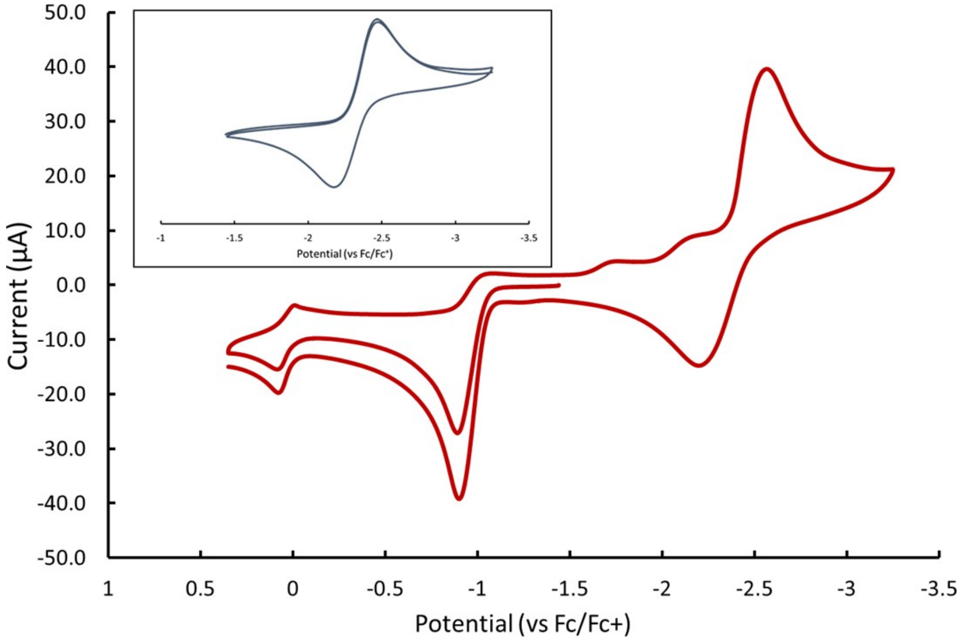

Figure S2. Cyclic voltammogram of **2**. (100 mV/s, 0.4 M NBu_4PF_6, THF). Inset shows the reversible reductive wave.

Details of Crystallographic Determination

Complex **1**: A colorless crystal (approximate dimensions 0.15 × 0.11 × 0.09 mm^3) was placed onto the tip of a 0.1 mm diameter glass capillary and mounted on a Bruker APEX II Kappa Duo diffractometer equipped with an APEX II detector at 150(2) K.

The data collection was carried out using Mo Kα radiation (graphite monochromator) with a frame time of 15 seconds and a detector distance of 7.00 cm. A collection strategy was calculated and complete data to a resolution of 0.82 Å with a redundancy of 4 were collected. Six sections of frames were collected with 0.50º ω and ϕ scans. The total exposure time was 21.42 hours. The frames were integrated with the Bruker SAINT[1] software package using a narrow-frame algorithm. The integration of the data using a monoclinic unit cell yielded a total of 55944 reflections to a maximum θ angle of 25.06° (0.84 Å resolution), of which 13672 were independent (average redundancy 4.092, completeness = 99.1%, R_{int} = 9.28%, R_{sig} = 9.29%) and 8589 (62.82%) were greater than $2\sigma(F^2)$. The final cell constants of a = 19.4894(10) Å, b = 16.2426(9) Å, c = 49.107(3) Å, β = 90.164(3)°, *volume* = 15545.2(14) $Å^3$, are based upon the refinement of the XYZ-centroids of 5128 reflections above 20 $\sigma(I)$ with 5.329° < 2θ < 49.52°. Data were corrected for absorption effects using the multi-scan method (SADABS).[2] The ratio of minimum to maximum apparent transmission was 0.883. The calculated minimum and maximum transmission coefficients (based on crystal size) are 0.9361 and 0.9610.

The space group C2/c was determined based on intensity statistics and systematic absences. The structure was solved and refined using the SHELX suite of programs.[3] An intrinsic methods solution was calculated, which provided most non-hydrogen atoms from the E-map. Full-matrix least squares / difference Fourier cycles were performed,

which located the remaining non-hydrogen atoms. All non-hydrogen atoms were refined with anisotropic displacement parameters. The hydrogen atoms were placed in ideal positions and refined as riding atoms with relative isotropic displacement parameters. The final anisotropic full-matrix least-squares refinement on F^2 with 937 variables converged at R_1 = 5.81%, for the observed data and wR_2 = 15.48% for all data. The goodness-of-fit was 1.035. The largest peak in the final difference electron density synthesis was 0.484 e-/Å^3 and the largest hole was -0.362 e-/Å^3 with an RMS deviation of 0.069 e-/Å^3. On the basis of the final model, the calculated density was 1.255 g/cm^3 and F(000), 6160 e$^-$. The structure was found as proposed with two crystallographically independent molecules per formula unit.

Complex **2**: A light brown crystal (approximate dimensions 0.046 × 0.036 × 0.005 mm^3) was placed onto the tip of an ultra thin glass rod and mounted on a D8 platform goniometer and measured at 100(2) K.

The data collection was carried out using synchrotron radiation (λ = 0.41328 Å, E =30 keV, diamond 111 monochromators, two mirrors to exclude higher harmonics) with a frame time 0.5 seconds and a detector distance of 5.0 cm. A randomly oriented region of reciprocal space was surveyed to the extent of a hemisphere. The total exposure time was 0.20 hours. The frames were integrated with the Bruker SAINT software package[1] using a narrow-frame algorithm. The integration of the data using a monoclinic unit cell yielded a total of 109505 reflections to a maximum θ angle of 16.93° (0.71 Å resolution), of which 22872 were independent (average redundancy 4.788, completeness = 98.4%, R_{int} = 6.23%, R_{sig} = 7.57%) and 14403 (62.97%) were greater than $2\sigma(F^2)$. The final cell constants of a = 24.9817(18) Å, b = 11.9639(9) Å, c = 26.6933(19) Å, β = 95.7654(13)°,

volume = 7937.7(10) Å^3, are based upon the refinement of the XYZ-centroids of 9969 reflections above 20 $\sigma(I)$ with 4.56° < 2θ < 32.92°. Data were corrected for absorption effects using the multi-scan method (SADABS[2]). The ratio of minimum to maximum apparent transmission was 0.901. The calculated minimum and maximum transmission coefficients (based on crystal size) are 0.9910 and 0.9990.

The space group $P2_1/c$ was determined based on intensity statistics and systematic absences. The structure was solved using and refined using the Shelx suite of programs.[3] An intrinsic methods solution was calculated, which provided most non-hydrogen atoms from the E-map. Full-matrix least squares / difference Fourier cycles were performed, which located the remaining non-hydrogen atoms. All non-hydrogen atoms were refined with anisotropic displacement parameters. The hydrogen atoms were placed in ideal positions and refined as riding atoms with relative isotropic displacement parameters. The final anisotropic full-matrix least-squares refinement on F^2 with 983 variables converged at R_1 = 6.46%, for the observed data and wR_2 = 19.35% for all data. The goodness-of-fit was 1.096. The largest peak in the final difference electron density synthesis was 0.947 e^-/Å^3 and the largest hole was -0.964 e^-/Å^3 with an RMS deviation of 0.242 e^-/Å^3. On the basis of the final model, the calculated density was 1.253 g/cm^3 and F(000), 3160 e^-. The structure was found as proposed with two crystallographically independent molecules per formula unit.

Table S1. Selected bond lengths (Å) and angles (°) for **1**.

Mn1A-C1A	2.151(4)	Mn1A-C25A	2.178(4)
Mn1A-C13A	2.179(4)	Mn1A-Cl1A	2.2831(12)
Mn1B-C25B	2.153(4)	Mn1B-C1B	2.177(4)
Mn1B-C13B	2.198(4)	Mn1B-Cl1B	2.2909(12)
C1A-Mn1A-C25A	88.69(14)	C1A-Mn1A-C13A	86.69(14)
C25A-Mn1A-C13A	86.34(14)	C1A-Mn1A-Cl1A	126.43(11)
C25A-Mn1A-Cl1A	124.96(10)	C13A-Mn1A-Cl1A	130.18(11)
C25B-Mn1B-C13B	85.45(14)	C1B-Mn1B-C13B	89.06(14)
C25B-Mn1B-Cl1B	133.50(12)	C1B-Mn1B-Cl1B	125.50(9)
C13B-Mn1B-Cl1B	122.54(11)		

Table S2. Selected bond lengths (Å) and angles (°) for **2**.

Mn1A-N7A	1.523(2)	Mn1A-C1A	1.938(2)
Mn1A-C25A	1.956(2)	Mn1A-C13A	2.006(2
Mn1B-N7B	1.519(2)	Mn1B-C1B	1.947(2)
Mn1B-C25B	1.968(3)	Mn1B-C13B	1.995(3)
N7A-Mn1A-C1A	121.39(11)	N7A-Mn1A-C25A	119.32(11)
C1A-Mn1A-C25A	90.75(9)	N7A-Mn1A-C13A	128.11(11)
C1A-Mn1A-C13A	97.01(9)	C25A-Mn1A-C13A	90.87(9)
N7B-Mn1B-C1B	116.14(12)	N7B-Mn1B-C25B	125.32(12)
C1B-Mn1B-C25B	91.23(10)	N7B-Mn1B-C13B	125.26(12)
C1B-Mn1B-C13B	91.29(10)	C25B-Mn1B-C13B	98.20(11)

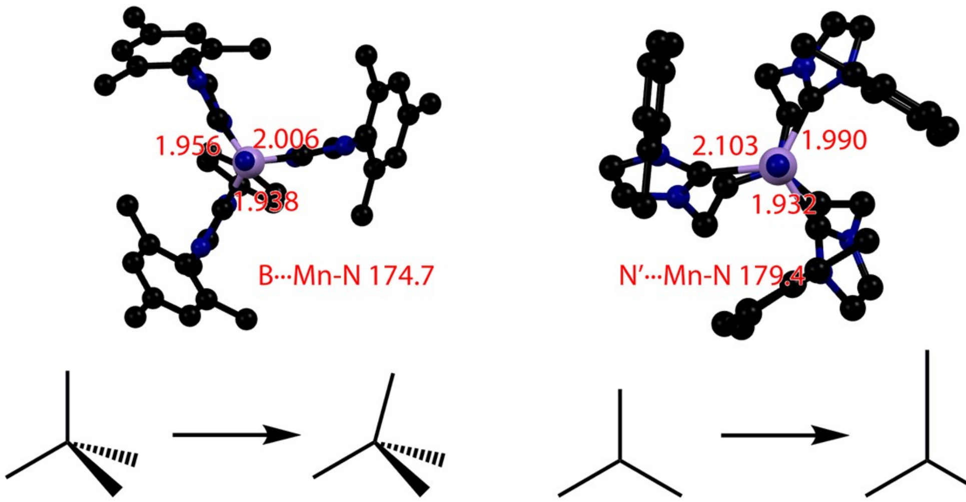

Figure S3. Illustration of the Jahn-Teller distortion in low spin Mn(IV) nitride complexes. Left: PhB(MesIm)$_3$Mn≡N (this work, **2**), where the Mn-C bond lengths are similar, but the B-Mn-N vector is bent. Right: [(TIMEN^{xyl})Mn≡N]$^+$, where the N'-Mn-N vector is linear, but one of the Mn-C bonds is elongated. Cartoon representations of the distortion are shown below the structures.

Table S3. Fitting parameters for the HR XPS Mn 2p spectra (Fig. 4 a,c)

	Band	Pos, eV	FWHM, eV	%Gauss	Area%	ChSquared
Complex **2**	1	641.54	2.97	100	55.62	1.28
	2	646.01	4.00	100	14.64	
	3	653.15	3.11	90	23.31	
	4	658.20	3.34	70	6.43	
Complex **1**	1	640.94	2.44	100	43.11	1.77
	2	645.28	3.92	80	21.58	
	3	652.98	3.24	90	23.45	
	4	658.63	3.34	70	11.86	

Table S4. Fitting parameters for the HR XPS Mn 3s spectra (Fig. 4 b,d)

	Band	Pos, eV	Pos. separation, eV	FWHM, eV	%Gauss	Area%	ChSquared
Complex **2**	1	83.41	5.30	2.82	80	50.30	1.05
	2	88.71		3.45	70	49.70	
Complex **1**	1	82.88	6.04	2.32	80	60.99	1.19
	2	88.86		2.45	74	39.01	

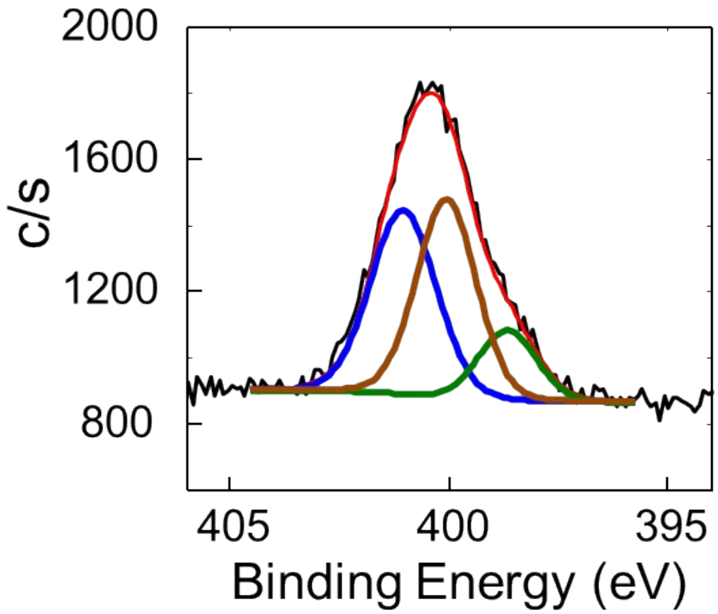

Figure S4. High-resolution N 1s spectra of complex **2**. The black line represents the experimental data, the red line shows the generated fit, and the blue, brown line and green line represent 3s split components. See Table S5 for fitting parameters.

Table S5. Fitting parameters for the HR XPS N 1s of complex **2** spectra (Fig. S4)

Band	Pos, eV	FWHM, eV	%Gauss	Area%	ChSquared
1	398.68	1.6	95	14.59	1.44
2	400.08	1.6	85	42.49	
3	401.07	1.78	90	42.92	

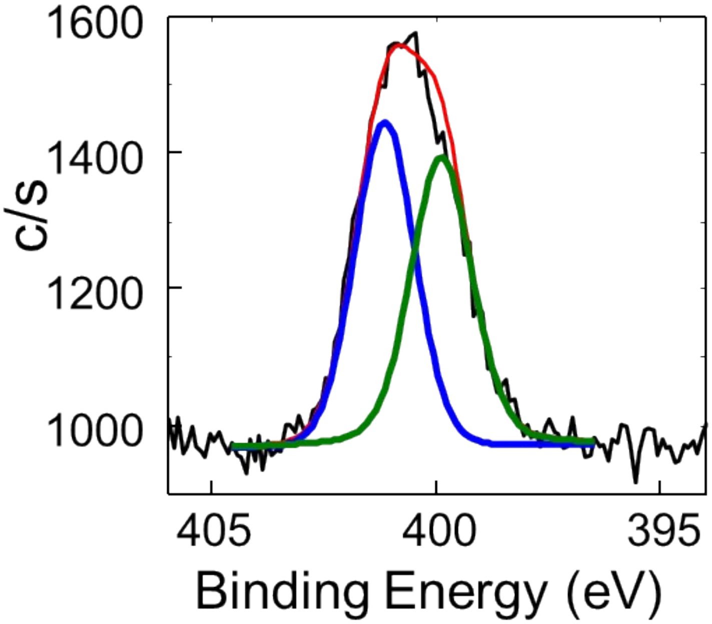

Figure S5. High-resolution N 1s spectra of standard sample. The black line represents the experimental data, the red line shows the generated fit, and the blue and green lines represent 3s split components. See Table S6 for fitting parameters.

Table S6. Fitting parameters for the HR XPS N 1s of standard sample spectra (Fig. S5)

Band	Pos, eV	FWHM, eV	%Gauss	Area%	ChSquared
1	399.89	1.6	75	49.43	1.35
2	401.16	1.6	95	50.57	

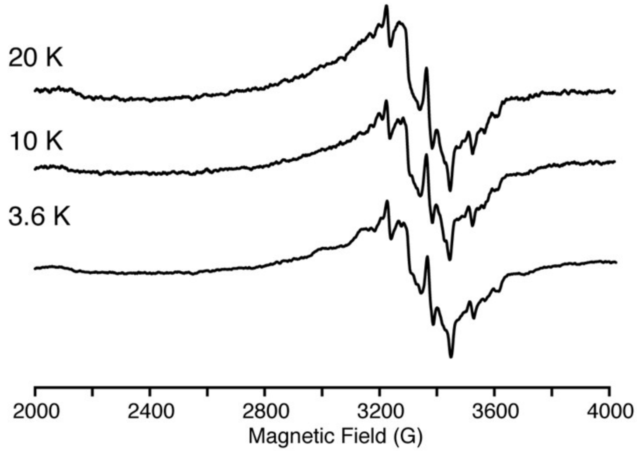

Figure S6. X-band (9.37 GHz) continuous-wave EPR of **2** as a suspended powder (slurry) at various temperatures indicated in the figure with 100 kHz field modulation (4 G modulation amplitude). A single S = ½ Mn(IV) complex is observed, identifiable by the ^{55}Mn hyperfine lines..

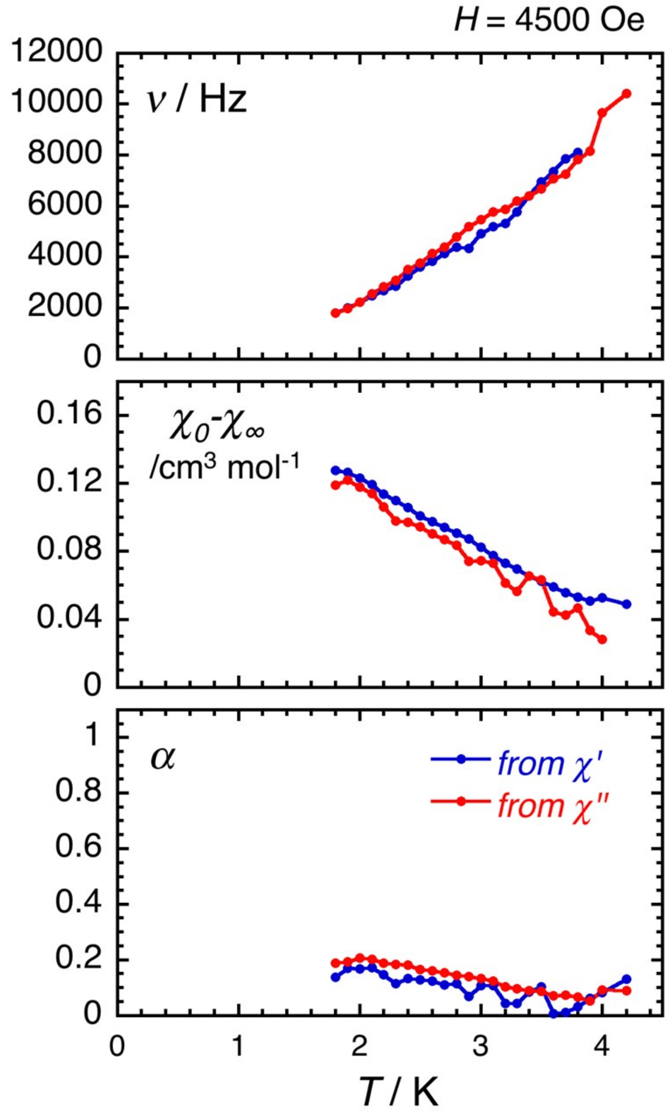

Figure S7. Temperature dependence of the magnetic parameters deduced from the fits of the in-phase (blue dots) and out-of-phase (red dots) ac susceptibility data shown in Figure 7 (right part) using a generalized Debye model (ν : characteristic ac frequency; χ_0- χ_∞ : amplitude of the relaxation mode with χ_0 and χ_∞ being the in-phase ac susceptibilities in the zero and infinite ac frequency limits, respectively; α : the distribution of the relaxation). The solid lines are guides for the eyes.

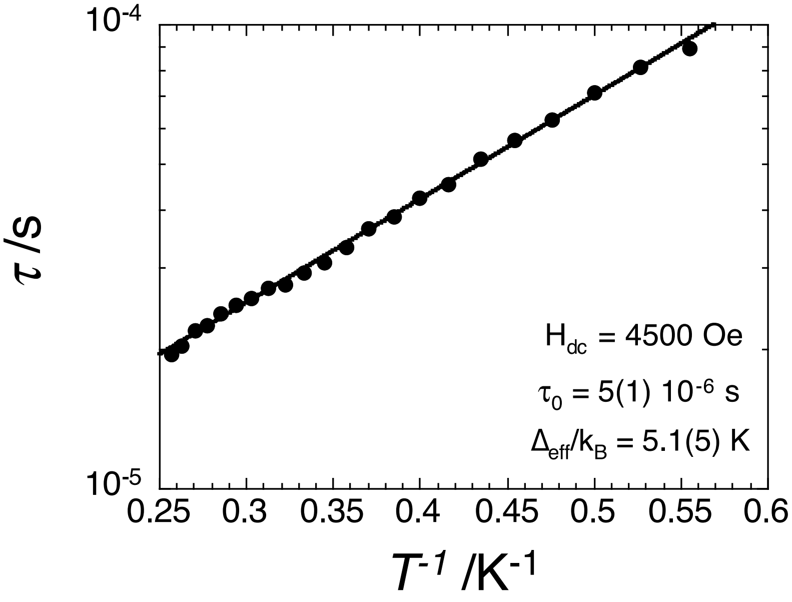

Figure S8. Temperature dependence of the relaxation time at 0.45 T shown in a semi-logarithm plot to emphasize the hypothetical thermally activated (Orbach) relaxation mechanism. The relaxation time was estimated by averaging time values calculated from the generalized Debye model applied to the frequency dependence of the in-phase and out-phase ac susceptibility shown in Figure 7 (see top part of Figure S7 for the individual characteristic frequency values). The solid lines is the best fit to the Arrhenius law.

References

[1]. SAINT, Bruker Analytical X-Ray Systems, Madison, WI, current version

[2]. An empirical correction for absorption anisotropy. R. Blessing, *Acta Cryst*. A51, 33 - 38 (1995).

[3]. A short history of *SHELX*. G. M. Sheldrick, *Acta Cryst*. A64, 112 - 122 (2008).
